# Comparative Study of Food-Grade Pickering Stabilizers Obtained from Agri-Food Byproducts: Chemical Characterization and Emulsifying Capacity

**DOI:** 10.3390/foods11162514

**Published:** 2022-08-20

**Authors:** César Burgos-Díaz, Yohanna Mosi-Roa, Mauricio Opazo-Navarrete, Mariela Bustamante, Karla Garrido-Miranda

**Affiliations:** 1 Agriaquaculture Nutritional Genomic Center, CGNA, Temuco 4780000, Chile; 2 Scientific and Technological Bioresource Nucleus (BIOREN), Department of Chemical Engineering, and Centre for Biotechnology and Bioengineering (CeBiB), Universidad de La Frontera, Temuco 4780000, Chile

**Keywords:** Pickering emulsions, agri-food byproducts, Pickering particles, emulsifying capacity

## Abstract

Natural Pickering emulsions are gaining popularity in several industrial fields, especially in the food industry and plant-based alternative sector. Therefore, the objective of this study was to characterize and compare six agri-food wastes/byproducts (lupin hull, canola press-cake, lupin byproduct, camelina press-cake, linseed hull, and linseed press-cake) as potential sources of food-grade Pickering stabilizers. The results showed that all samples contained surface-active agents such as proteins (46.71–17.90 g/100 g) and dietary fiber (67.10–38.58 g/100 g). Canola press-cake, camelina press-cake, and linseed hull exhibited the highest concentrations of polyphenols: 2891, 2549, and 1672 mg GAE/100 g sample, respectively. Moreover, the agri-food byproduct particles presented a partial wettability with a water contact angle (WCA) between 77.5 and 42.2 degrees, and they were effective for stabilizing oil-in-water (O/W) emulsions. The emulsions stabilized by Camelina press-cake, lupin hull, and lupin by-product (≥3.5%, *w*/*w*) were highly stable against creaming during 45 days of storage. Furthermore, polarized and confocal microscopy revealed that the particles were anchored to the interfaces of oil droplets, which is a demonstration of the formation of a Pickering emulsion stabilized by solid particles. These results suggest that agri-food wastes/byproducts are good emulsifiers that can be applied to produce stable Pickering emulsions.

## 1. Introduction

Every year, the food industry generates large amounts of waste or byproducts (billions of tons) from different sources, which in many cases are simply discarded by the industry concerned [1]. These wastes/byproducts are an excellent source of valuable compounds, such as polysaccharides, proteins, fats, fibers, antioxidants, natural emulsifiers, and bioactive compounds, which may be utilized in different industrial applications due to their nutritional and techno-functional properties [2,3]. Accordingly, there is a large opportunity and challenge to valorize these byproducts, and thus contribute to the circular economy and environmental protection. Therefore, it is necessary to find specific and relevant solutions to valorize these food wastes/byproducts in innovative applications. In this perspective, an interesting aspect is the utilization of agri-food wastes/byproducts as an alternative source, renewable, and inexpensive source of “natural stabilizers” (solid amphiphilic particles) to develop food-grade Pickering emulsions. It should be noted that the development and studies in this area are still very limited.

In recent years, the investigation and utilization of Pickering emulsions have attracted significant interest in the research field of foods and pharmaceutics [4]. In these colloidal suspensions, stabilization is achieved by using only solid particles (hence the alternative name of solid-stabilized emulsions) in the place of organic surfactants and polymers [5]. Pickering emulsions display several advantages over conventional surfactant-stabilized emulsions, such as high stability against coalescence (even when the droplets are large) and Ostwald ripening, the emulsions are surfactant-free, and others [6]. Additionally, Pickering emulsions seem to be much more advantageous and promising for developing encapsulation and delivery systems for bioactive compounds in contrast with conventional emulsions [7]. 

Natural Pickering particles are preferred by the food or pharmaceutical industries because of their noteworthy natural benefits (renewable resources, ease of preparation, excellent biocompatibility, and unique interfacial properties) [8]. To date, several studies have addressed particle-stabilized Pickering emulsions. However, a limited number of these studies are directly compatible with foods, since most of the particles (especially inorganic particles) used for Pickering stabilization are not food-grade [4]. On the other hand, there is only a limited number of inorganic particles that are permitted in food, pharmaceutical, and cosmetic applications due to biocompatibility and biodegradability issues [9]. Moreover, some of these particles require substantial effort to synthesize because of the time-consuming, expensive, and unsustainable processes involved [10]. Accordingly, in recent years, a large number of natural, food-grade, and biocompatible particles have been discovered or developed, which can be divided into seven categories: polysaccharide particles (starch, chitosan, cellulose), fat crystals [11,12], complex particles [13], flavonoid particles [14], food-grade wax [15], protein-based particles proteins (zein, whey, soy, lupin) [4,16,17,18], and byproducts (apple peel pomace, pomace, cocoa, and rapeseed cake) [19,20,21]. 

Accordingly, this study proposes to evaluate effective and cheap solid amphiphilic particles derived from natural sources to formulate stable Pickering emulsions. Moreover, the use of novel particles from “vegetable sources” would respond to consumer demand for a replacement of synthetic molecules with environmentally friendly compounds and also the valorization of wastes/byproducts from the agroindustry. In this context, the use of byproducts derived from natural sources such as oil seeds and legume grains has attracted great interest due to their techno-functional properties and low cost [22]. These agri-food wastes are highly nutritional and contain high-value functional ingredients, such as proteins, polysaccharides, fibers, flavor molecules, and phytochemicals [20,23]. Therefore, this research had the challenge of exploiting alternative “surface-active agents” derivatives from vegetable sources. Thus, the functionality of different types of powdered byproducts, as potential Pickering stabilizers, were evaluated and compared: (i) lupin hulls, (ii) linseed hulls, (iii) canola press-cake, (iv) camelina press-cake, (v) linseed press-cake, and/or (vi) byproduct from lupin protein isolation process. These natural sources were selected because they are an abundant byproduct of the agri-food industry, and also for their chemical composition (insoluble proteins, amphiphilic agents, fibers, among others). These characteristics make them good candidates for their use as Pickering stabilizers. For instance, lupin hull (30% of seed weight) is generally removed from the seed for improving the nutritional value of lupin grit, thus yielding a valuable byproduct [24]. The insoluble proteins and fibers present in the lupin hull could be strongly adsorbed at the oil-water interface, forming a strong barrier against droplet coalescence in the emulsion. Therefore, lupin hull powder is a good alternative as a Pickering stabilizer agent due to the presence of surface-active compounds. Regarding oilseed press-cake, these byproducts are generated from the production of vegetable oilseeds such as canola, camelina, rapeseed, flaxseed, soybeans, and sunflower seeds [25]. Therefore, prompt attention is required to handle these byproducts/wastes and reuse them as value-added ingredients or techno-functional materials for the food industry. 

On the other hand, during the protein isolation process from lupin (Alu*Prot*-CGNA^®^), different byproducts are generated with valuable compounds [24]. Specifically, in the technological process for obtaining this protein-rich functional ingredient, a valuable byproduct based on insoluble fiber-protein compounds is generated with potential surface-active properties. Therefore, here there is an opportunity to assess this insoluble protein-rich byproduct to potentially stabilize food-grade Pickering emulsions. 

Based on the above considerations, this research aimed to compare the effectiveness of six “vegetable amphiphilic solid particles” obtained from agri-food wastes/byproducts in the stabilization of O/W Pickering emulsions. Therefore, physicochemical characterization of each agri-food byproduct was evaluated. Then, O/W Pickering emulsions were obtained and characterized in terms of the droplet size, microstructure, and stability.

The success of this research will allow valorizing wastes/byproducts derived from the agri-food industry through a facile and cost-effective solution, thereby contributing to the circular economy. Finally, the results from this research proposal will provide a better understanding of the application of natural Pickering particles to stabilize O/W emulsion.

## 2. Materials and Methods

### 2.1. Materials

In this study, six powdered byproducts as potential Pickering stabilizers (lupin hull, lupin byproduct, linseed press-cake, linseed hull, camelina press-cake, and canola press-cake) were evaluated and provided by Agriaquaculture Nutritional Genomic Center (CGNA). The sunflower oil was acquired from a local supermarket (Temuco, Chile). The sodium acetate buffer, sodium dodecyl sulfate (SDS), and sodium azide were acquired from Sigma-Aldrich (Saint Louis, MO, USA).

### 2.2. Treatment of Agri-Food Byproducts

In this study, the agri-food byproducts/wastes were treated to obtain a fine powder (Pickering stabilizers). Therefore, to reduce particle size, all samples were milled using a rotor mill (Fritsch Mill Pulverisette 14, Indar-Oberstein, Germany) and sieved through a 106 μm aperture mesh to obtain a fine powder as a Pickering stabilizer. The camelina and canola linseed press-cake powders were defatted with hexane before grinding. 

The term “Pickering solid particles” refers to the fraction of the powder that is insoluble in water. Therefore, in this study only “the insoluble fraction” of each powdered byproduct was used to stabilize the O/W emulsions.

### 2.3. Powder Wastes/Byproducts Characterization

#### 2.3.1. Proximate Composition of Powder Wastes/Byproducts 

The protein content was determined by the Dumas method (Dumatherm^®^ N PRO analyzer, Königswinter, Germany) using a conversion factor of 6.25 to convert nitrogen values to protein (AOAC Official Method 930.03). The ash content was gravimetrically determined after heating at 550 °C in a muffle furnace (the AOAC Official Method 942.05). Moisture was determined by the oven method at 105 °C and measured using the gravimetric method (NCh 841 Of.78). The oil content was extracted with petroleum ether in a Soxhlet system (AOAC Official Method 920.39) and determined by a gravimetric method. The total dietary fiber was determined by the enzymatic gravimetric method (AOAC Official Method 985.29) [26], and, finally, carbohydrates (nitrogen-free extracts) were calculated by the difference. Results are expressed on a dry weight basis.

#### 2.3.2. Determination of Soluble and Insoluble Fraction

For the analysis of soluble and insoluble fractions, 5% (*w*/*w*) of the agri-food byproduct samples were dispersed in deionized water and stirred by using a multivortex for 20 min. After that, samples were centrifuged for 1 h at 10,000× *g* (Labogene 1580R, Gyrozen Co., Ltd.a., Daejeon, Korea). The supernatant containing the soluble fraction and pellet (water-insoluble fraction) was collected and the soluble and insoluble (%) of the samples were calculated by gravimetry. 

#### 2.3.3. Size and Zeta Potential Measurements 

The particle size, zeta-potential, and polydispersity index of each agri-food byproduct dispersion (1% *w*/*v* of sample in deionized water) were measured by using a Nanotrac Wave II analyzer (Microtrac MRB, Montgomeryville, PA, USA) at 25 °C. Thus, 0.5 mL of each byproduct was added to the cuvette of the analyzer for its analysis. A refractive index of 1.45 was used for all samples. 

#### 2.3.4. Total Polyphenols Content

Total phenolic content was determined by the Folin–Ciocalteau method following described by Opazo-Navarrete et al. [27]. In brief, 0.5 g of each byproduct was mixed with 20 mL of ethanol (1:40, *w*/*v*). Later, 50 μL of extracts was mixed with 50 μL Folin–Ciocalteau (FC) reagent (FC: distilled water, 1:10) and was left to stand for 5 min; 100 μL of Na_2_CO_3_ (0.2 g/L) and 800 μL of distilled water was added to the mixture. Afterwards, the samples were then heated at 45 °C for 15 min in a water bath. The mixtures were shaken and maintained in the dark for 30 min. Then, the absorbance was measured using a Multi-Detection Microplate Reader (BioTek SynergyTM HTX, Winooski, VT, USA) at 750 nm. The total phenolic content was expressed as mg of gallic acid equivalents (GAE) per gram of dry weight sample (mg GAE/100 g sample).

#### 2.3.5. DPPH Radical Scavenging Ability

The DPPH radical scavenging activity was performed according to Burgos-Díaz et al. [28]. An aliquot of 0.5 mL of each sample dispersion (0.5–20 µg/mL of powder agri-food byproduct) was mixed with 0.5 mL of DPPH (0.1 mM) in ethanol−95%. The mixture was shaken thoroughly and kept in darkness at room temperature for 30 min. The absorbance was measured using a Multi-Detection Microplate Reader (BioTek Synergy^TM^ HTX, Winooski, VT, USA) at 517 nm. Ascorbic acid was used as a positive control. The DPPH radical scavenging effect was calculated as follows:

DPPH radical scavenging effect (%) = (1 − A_1_/A_0_) × 100 (1)

where A_0_ is the absorbance of the control (distilled water) and A_1_ is the absorbance of the sample.

The IC_50_ DPPH values (the concentration of sample required for inhibition of 50% of DPPH radicals) were obtained through linear regression to calculate the dose/concentration required for a 50% reduction of the DPPH radical.

#### 2.3.6. Water Contact Angle (WCA) Measurements

WCA measurements of each powdered agri-food by-product were performed according to Burgos-Díaz et al. [4]. Briefly, a thin film of each sample was prepared by adding 1% (*w*/*v*) of dispersion (sample in distilled water) on a clean glass slide and then left to dry at 65 °C for 10 min. A drop of de-ionized water (1–2 μL) was then deposited on the surface of the film, and then the WCA (between the water drop and the particles film) was determined. Images of water droplets were obtained with a modular stereo microscope (Leica MZ10 F, Nanterre, France). The WCA was calculated by using a Gimp image manipulation software. The values correspond to an average of at least three drops.

#### 2.3.7. Microstructure Observation of Pickering Particles

The particles’ microstructure was evaluated by light microscopy using optical microscopy (Olympus-BX40, Tokyo, Japan) equipped with a camera and polarized light filters to visualize the crystal structures of plant particles. For this, a small volume of each sample was deposited on a microscopy glass slide and covered with a cover slip.

### 2.4. Pickering Emulsion Preparation

The emulsions were prepared according to Joseph et al. [20] with some modifications. Thus, each powdered agri-food byproducts concentration used varied from 1 to 5% (*w*/*w*), and the sunflower oil content was 20% (*w*/*w*). The pH of the aqueous phase was fixed at 7 using a buffer phosphate solution. A pre-emulsion was first obtained through “high-speed homogenization” followed by a microfluidization. Thus, with the Ultra-Turrax, each byproduct powder was dispersed in the buffered solution with agitation for 20 min, before being processed in an Ultra-Turrax mixer (Kinematica, Polytron© PT2500E, Luzern, Switzerland) at 6000 rpm for 2 min. The speed was then increased to 8000 rpm and the oil phase was progressively added to the water phase (2 min). Stirring was prolonged for 10 min at 12,000 rpm. Then, the system was subjected to microfluidization and homogenized at a pressure of 600 bars using a high-pressure homogenizer (GEA Lab Homogenizer Panda PLUS 1000, Parma, Italy). The emulsion was submitted to 3 passes through the chamber. Finally, the obtained emulsions were stored at 4 °C after preparation.

### 2.5. Pickering Emulsion Characterization

#### 2.5.1. Microstructure Observation of Pickering Emulsions

The microstructure of Pickering emulsions stabilized with the different agri-food byproducts was visualized using an optical microscope (Olympus-BX40, Tokyo, Japan) equipped with a camera to estimate the droplet size and aggregate state. The confocal laser scanning microscopy (Olympus Fluoview 1000, Tokyo, Japan) was used to assess the interfacial structure of Pickering emulsions. Thus, the Pickering emulsions were dyed with rhodamine B before the emulsion preparation. The pictures of the samples were acquired using the FV-ASW (v. 1.7) software. 

#### 2.5.2. Droplet Size Measurements

According to Joseph et al. [20], for emulsion droplet size measurements, a specific treatment with SDS was applied to the Pickering emulsions to eliminate particles present both in the bulk phase and at the oil/water interface. For this, the emulsions were diluted with 10% SDS in a 1:4 ratio. The system was then stirred with a magnetic bar for 7 h. The emulsions (diluted in the SDS solution) were centrifuged for 5 min at 220× *g* to separate the particles (sediment) from the oil droplets (cream). The creams were collected and diluted with phosphate buffer in 1:50 ratios and analyzed. The size distributions were measured using the Nanotrac Wave II Model (Microtrac MRB, USA). The droplet refractive index used was 1.47 and the aqueous phase was 1.33. Each measurement was performed in triplicate. The droplet’s distributions were described in terms of their volume-averaged diameter.

#### 2.5.3. Creaming Index of Emulsions

The emulsion stability of the Pickering emulsion was analyzed through the evolution of the creaming index (CI) for 45 days following the methodology described by Burgos-Díaz et al. [4]. Briefly, 5 mL of each O/W emulsion was deposited into a glass tube and then sealed to prevent moisture evaporation. All samples were monitored for 45 days. CI percentage (%) values were determined by using the following equation:

CI (%) =(Hs/He) × 100(2)

where Hs correspond to the serum layer height, and He is the total emulsion height.

### 2.6. Statistical Analysis

The analyses were performed in triplicate and the values were expressed as means ± mean deviations. All statistical analyses were performed by ANOVA and post hoc Tukey’s HSD using R freeware through the Rstudio interface.

## 3. Results and Discussion

### 3.1. Agri-Food Byproducts Particle Characterization

Agri-food byproducts represent a potential and cheaper source of macronutrients such as proteins, dietary fiber, carbohydrates, and phenolic compounds, among others. Proximate analysis of each byproduct is shown in Table 1. All samples contain valuable macronutrients, such as proteins, carbohydrates, ash, dietary fiber, and fat. Regarding protein content, Camelina press-cake contains the highest concentration (46.71 g/100 g), followed by linseed press-cake (40.97 g/100 g), canola press-cake (39.80 g/100 g), lupin byproduct (30.80 g/100 g), linseed hull (22.97 g/100 g), and lupin hull (17.90 g/100 g). The presence of proteins (soluble and insoluble) is relevant due to the fact that they are the most important surface-active compounds in plant materials. It is also important to highlight the high dietary fiber content in lupin hull (67.10%), linseed hull (61.10%), and lupin byproduct (60.59%). While the canola, linseed, and camelina press-cake presented a value close to 40% of dietary fiber. Different studies have shown the potential of fibers as a Pickering stabilizer to prepare O/W emulsions. For instance, water-insoluble dietary fibers from the bamboo shoot were used as plant food particles for the stabilization of O/W Pickering emulsions [29], and citrus fiber-stabilized emulsions had excellent stability against various conditions [30].

On the other hand, all byproducts contain a wide percentage of insoluble fractions with values between 71% and 83% (Table 2). This is important since the term “Pickering particles” refers to the fraction of the powder that is insoluble in water. The powder particles have an irregular form and mean size lower than 5 µm. The zeta-potential of agri-food particles ranged from 25.13 mV to 37.53 mV (absolute value) with linseed hull and camelina press-cake, respectively. These results indicate that agri-food byproducts particles have a negative surface charge when they are dispersed in water at pH 7. According to Tavares et al. [31], the zeta-potential values higher than 30 mV or less than −30 mV indicate a stable dispersion due to electrostatic repulsion. In this sense, the particle dispersions (agri-food byproducts in the water) could be considered stable systems due to their zeta-potential values being close to 30 mV (absolute value).

On the other hand, the polydispersity index (PDI) was used to describe the degree of uniformity of the size distribution of agri-food byproducts particles. In this sense, a sample is considered monodisperse when the PDI value is less than 0.1 [32]. Accordingly, all our particles are polydisperse, except for camelina press-cake (PDI: 0.08).

### 3.2. Antioxidant Activity of Agri-Food Byproduct Particles

**Figure 1:**
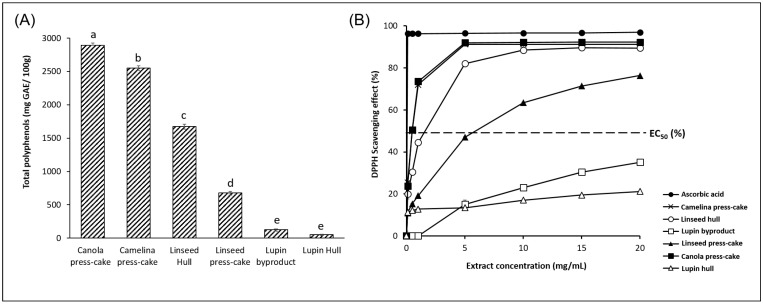
(**A**) Total polyphenol content of the agri-food byproducts. Different letters in the bars indicate a significant difference (*p* < 0.05) between samples. (**B**) DPPH radical scavenging effect of the agri-food byproducts.

Different groups of phenolic compounds, carotenoids, vitamins, bioactive polysaccharides, and dietary fibers are some of the examples of potentially bioactive compounds found in plant-based byproducts [33]. These compounds are plant-based molecules that have recognized benefits in human health as potent antioxidants and anti-inflammatory agents [27]. Figure 1A shows the total polyphenols content of the six agri-food byproducts evaluated in this study. The results showed that the polyphenol contents of canola press-cake, camelina press-cake, linseed hull, linseed press-cake, lupin byproduct, and lupin hull decreased sequentially to 2891.0, 2549.6, 1672.6, 680.8, 127.8, and 55.0 mg GAE/100 g.

On the other hand, the plant-based byproducts showed different scavenging abilities in a dose-dependent way (Figure 1B). The DPPH scavenging ability obtained for agri-food particles ranged from 0% to 90% at extract and increased from 0.5 to 12.5 µg/mL. Mention that the values obtained at high concentrations are very close to the value for ascorbic acid (positive control).

DPPH scavenging effect followed the same trend as the total polyphenol content, which would indicate that polyphenols are the compounds responsible for the antioxidant activity of the six agri-food byproducts.

The effective concentration for 50% scavenging of DPPH radicals (EC_50_) was 0.500 mg/mL for camelina press-cake, 0.821 mg/mL for canola press-cake, 1.575 mg/mL for linseed press-cake, and 5.877 mg/mL for linseed hull, while for lupin hull and lupin byproduct, the values were lower than EC_50_. This could be due to a low lutein content found in the lupin hull sample. Regarding canola and camelina press-cake, oilseed cakes are generally rich in oleochemicals, and phytochemicals with antioxidant activity [34], which could have influenced the scavenging of DPPH radicals and lower EC_50_ values obtained. 

It is important to highlight that high values of antioxidant activity and polyphenol content in these byproducts could help to reduce the oxidation rate of the oil [21,35] or of the bioactive compound contained in the oil phase of the emulsion, since this type of by-product works as a protective layer of the oil against oxidative deterioration, granting greater oxidative stability [36].

### 3.3. Wetting Properties of Pickering Stabilizers

**Figure 2:**
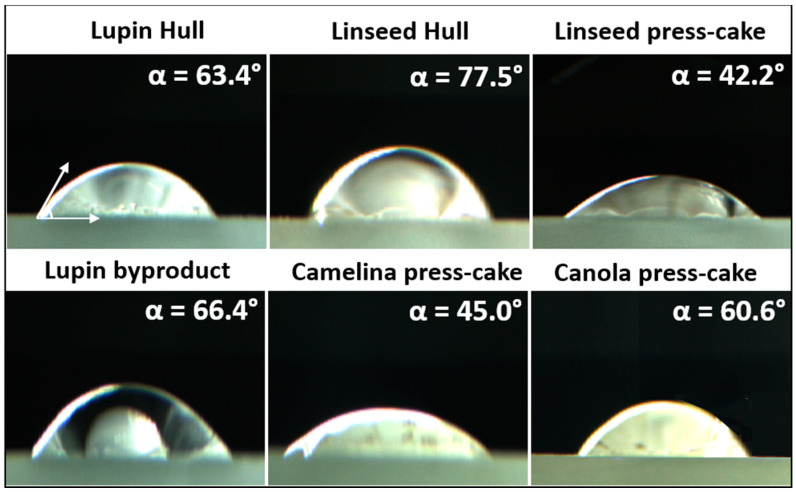
Water contact angles of each byproduct particle film.

The wettability of each agri-food particle was qualitatively estimated from the water contact angle (WCA) measurements (calculated from captured pictures, Figure 2). Thus, based on how the water drop interacts with the solid surface, the particles can be classified as hydrophilic or hydrophobic. As can be seen from Figure 2, all samples (agri-food byproducts particles) exhibited a WCA smaller than 90°, showing their hydrophilic character. The linseed hull particles showed the highest WCA value (θ = 77.5°), while linseed press-cake presented the lowest value (θ = 42.0°). These results are in agreement with the results obtained by Lu et al. [37], in which a food-grade Pickering stabilizer presented a hydrophilic character with a contact angle of ~55°, having also an excellent emulsifying property to stabilize the O/W emulsion. In general, particles with a contact angle in the range of 15° < θ < 90° should stabilize better O/W emulsions [5]. The formulation of Pickering O/W emulsions is strongly influenced by the hydrophobicity of the agri-food particles, which depends on the oil-water interface contact angle. According to the Bancroft rule, hydrophilic particles (i.e., with a contact angle <90° measured through the water phase) are better for stabilizing O/W emulsions. Conversely, hydrophobic particles (i.e., with a contact angle >90°) are more suitable for stabilizing W/O emulsions [35].

In this study, interestingly, no relationship was observed between the wettability (WCA values) of byproducts and their emulsifying capacity. For instance, camelina press-cake showed a remarkable emulsifying capacity at a concentration of ≥2.5%, *w*/*w* (data shown in Section 3.4.3), which is somewhat inconsistent with the result of wettability (WCA equal to 45°). Theoretically, the contact angle around 90° is preferable for stabilizing Pickering emulsions. However, the formation of Pickering emulsion can be also influenced by many other factors, such as particle size, electrical potential, and particle shape [21], among others. According to the study by Lu et al. [21], the reduction of WCA from 109.7° to 66.4° of an apple byproduct positively affected the formation of an oil-in-water emulsion. In addition, Lu et al. [37] showed that the WCA of starch particles was 55° and that it also had excellent emulsifying properties for stabilizing O/W Pickering emulsion. 

### 3.4. Pickering Emulsions Characterization

#### 3.4.1. Influence of Agri-Food Byproduct Concentration on Emulsion Droplet Size

**Figure 3:**
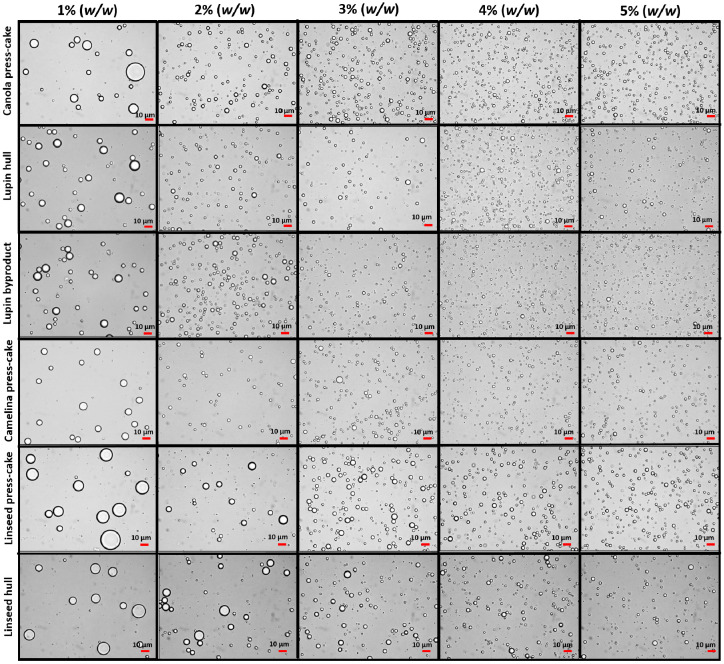
Optical micrographs of the O/W Pickering emulsions stabilized at different byproduct concentrations (1.0–5.0%, *w*/*w*). The images were acquired at 40× magnification. The emulsions were prepared at a fixed oil concentration (20%, *w*/*w*).

Figure 3 showed that the droplet size of the O/W emulsions with different agri-food particle concentrations changed with increasing particle percentages, from 1 to 5% (*w*/*w*) and at a fixed oil concentration (20%, *w*/*w*). In general, the droplet size was much smaller at high particle concentrations than for those emulsions stabilized at low particle concentrations, confirming an improvement in the Pickering emulsification performance of the particles from agri-food byproducts. Moreover, the microscopy micrographs also show that the emulsion droplet size varied among the different types of agri-food byproducts. 

**Figure 4:**
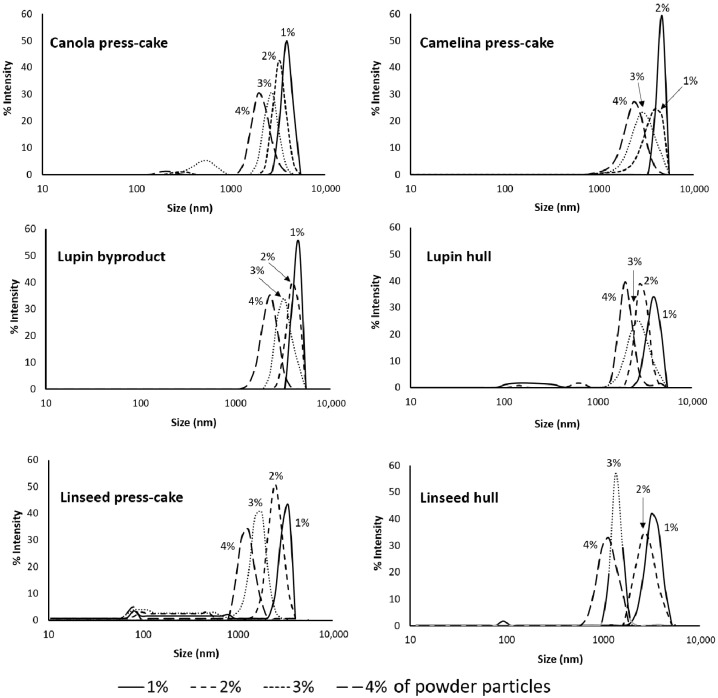
Particle size distribution graphs of the emulsions (O/W) stabilized with different concentrations of powder byproduct (1.0–4.0%, *w*/*w*).

On the other hand, Figure 4 showed that the particle size distribution of the droplet of emulsions (O/W) is rather similar among all emulsions stabilized with different byproducts, where the size distribution peaks decrease with increasing the particle concentration. Thus, the emulsions presented a monodispersed particle distribution, where the mean particle size was dependent on the byproduct concentration. It should be noted that the particle size distribution for emulsions stabilized with a 5% (*w*/*w*) of particles showed similar behavior to those stabilized by 4% (*w*/*w*) (data not shown).

Accordingly, as can be observed in Figure 3 and Figure 4, if the concentration value is low, the oil droplets’ size is much larger, which is attributed to the number of particles, which tends to not be enough to completely cover the interface of newly formed oil droplets in the homogenization process. Therefore, coalescence and even oiling off can occur in the emulsion [7]. In contrast, at high concentrations, the surface coverage could be enough to produce smaller droplets. It is important to mention that to form an effective barrier against coalescence, Pickering particles should not only be able to adsorb at the oil-water interface, but also to completely cover the oil droplets [38]. Frelichowska et al. [39] and Burgos-Díaz et al. [4] observed that with the increase in particle concentration, smaller droplets were obtained with improved O/W Pickering emulsion stability. Therefore, the Pickering emulsion droplet size and the resistance to coalescence depend on the agri-food particle concentration. 

#### 3.4.2. Emulsion Microstructure Characterization 

**Figure 5:**
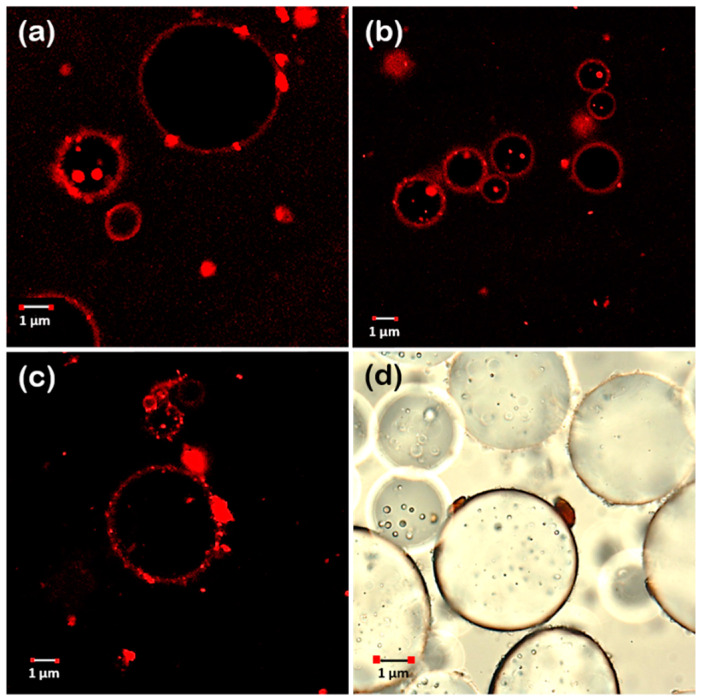
Pictures (**a**–**c**) correspond to CLSM micrographs of the O/W emulsions stabilized with byproducts. (**d**) Representative micrographs of the particles anchored to the interfaces of oil droplets. The particles correspond to lupin hull, camelina press-cake, and canola press-cake.

Figure 5a–c show the CLSM (confocal laser scanning microscopy) images of each particle-stabilized emulsions stained with Rhodamine B fluorescent dye (protein marker), which was chosen because proteins are considered the main surface-active compounds responsible for Pickering emulsion stabilization [40]. The images clearly showed droplets surrounded by a red bright ring, which indicated that oil droplets were covered with a dense layer of accumulated particles, which correspond to agri-food byproducts stabilizers. According to Burgos-Díaz et al. [4], this behavior is a clear confirmation that O/W emulsions are stabilized by Pickering solid particles. 

**Figure 6:**
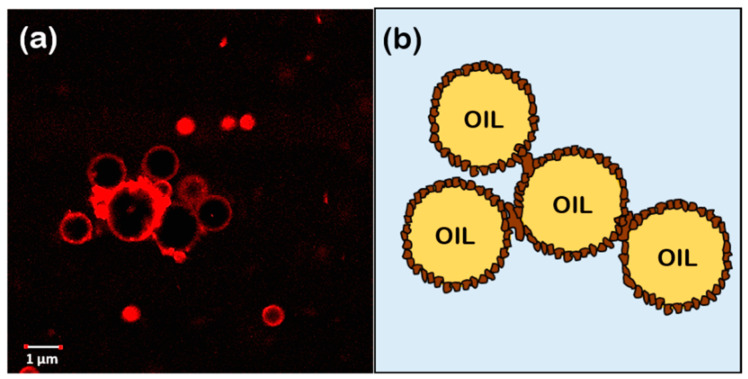
Picture (**a**) corresponds to CLSM micrographs of an emulsion stabilized by lupin hull. (**b**) Schematic representation of the oil droplet distribution.

In addition, a droplet–droplet bridging was observed (see Figure 6) due to particles simultaneously adsorbed at the surface of two neighboring droplets, which is only possible when the number of particles may not be sufficient to completely cover the oil-interface droplets, and, consequently, two neighboring droplets can share the same Pickering particle, forming a bridge between the droplets. According to Schröder et al. [38], in O/W emulsions, bridging can occur when particles have dual wettability; however, they are still largely hydrophilic, i.e., the phase contact angle is considerably smaller than 90°. This is in agreement with the value calculated for our particles based on angle contact images (Figure 2). Moreover, agri-food byproducts particles were relatively large (higher than 0.1 μm) and they can attach two or more oil droplets on their periphery. This bridging configuration favors the formation of large droplet flocs. The bridges can prevent the droplet–droplet coalescence, and emulsion stability by increasing the rate of flocculation and creaming.

On the other hand, the images revealed that oil droplets form large and dense flocs, which are immobilized. According to Schröder et al. [38], this behavior can be attributed to the fact that particles may form a three-dimensional network of aggregated particles in the continuous phase of the emulsion, which enhances emulsion physical stability.

**Figure 7:**
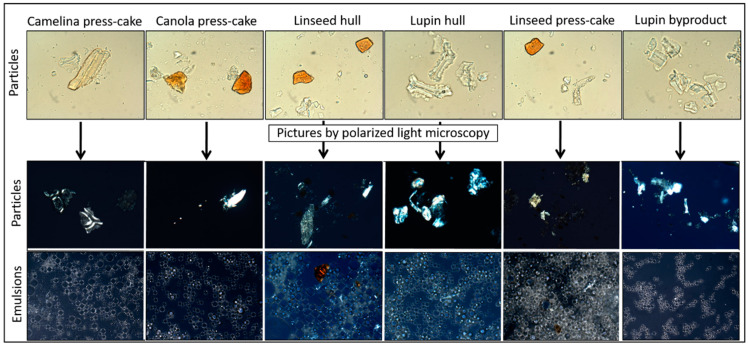
The upper micrographs correspond to the powder dispersions. The middle and lower pictures correspond to polarized light microscopy images of particles and emulsions produced by powder byproducts (particles). The images were acquired at 40× (particles) and 100× (emulsions) magnification.

In addition, a distinctive birefringent polarization ring was visualized from polarized light microscopy images (Figure 7), indicating that particles adsorb at the oil–water interface and retain their crystallinity. In addition, the polarization ring was brighter with some types of particles, indicating increasing amounts of particles adsorb at the interface, while substantial amounts of particles remain in the continuous phase as is clear from the continuous phase polarization. This crystalline shell around the oil droplets (bright ring) was not only in freshly prepared emulsions (data not shown). Similar results were visualized by Schröder et al. [38,41], who observed a “bright ring” at the emulsion droplet surface by polarized light microscopy, which indicated that the particles are present at the oil–water interface.

#### 3.4.3. Evolution of Emulsion Stability in the Time

**Figure 8:**
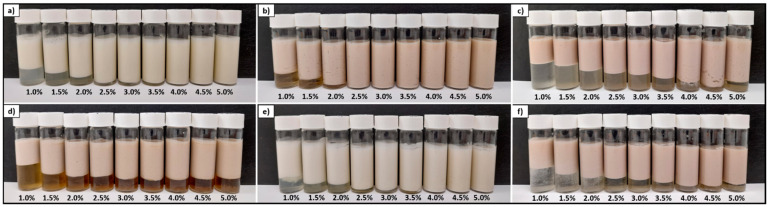
Images of the O/W emulsions stabilized by powder byproducts at different concentrations (1–5%, *w*/*w*) after storage for 45 days. (**a**) lupin hull; (**b**) camelina press-cake; (**c**) linseed press-cake; (**d**) canola press-cake; (**e**) lupin byproduct; (**f**) linseed hull.

The emulsion stability was evaluated by the creaming index (CI) and visual observation of Pickering emulsions. Mention that creaming is induced by density differences between the dispersed phase (oil) and the continuous phase (water). Thus, the creaming destabilization leads to an emulsion phase separation, shown by a clear phase at the sample bottom tube known as clarification [3]. As shown in Figure 8, for the emulsions stabilized by lupin byproduct and lupin hull at a concentration of ≥3.5% (*w*/*w*), no creaming behavior was detected after 45 days, and therefore there can be said to have been a major stability during the storage period. While for camelina press-cake this behavior was observed with a lower concentration of particles (≥2.5%, *w*/*w*). These observations indicated that the type of sample and increasing the concentration of the particles gradually improved the emulsion stability in this type of sample. In addition, all emulsions (visually, Figure 8) were stable against coalescence since no macroscopic oil leakage was observed at the top of the tubs. It is worth mentioning that, because of the strong aggregated state, some emulsions were highly viscous (e.g., emulsions stabilized by camelina press-cake), even gelled in some cases, which delayed gravity-driven phenomena. 

**Figure 9:**
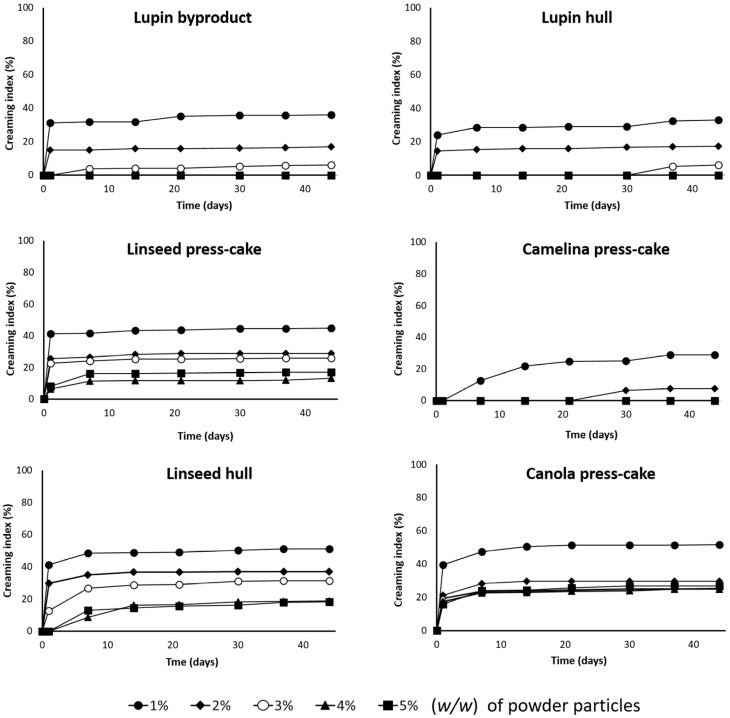
Evolution of CI (%) of the emulsions stabilized by agri-food byproduct particles at different concentrations (1–5%, *w*/*w*) over 45 days.

Figure 9 shows the evolution of the CI (%) of all emulsions at different particle concentrations (from 1 to 5%, *w*/*w*) and during 45 days of storage. As was expected, the creaming behavior of the emulsions was strongly influenced by the particle concentration and type of sample. This is in agreement with the results observed in Figure 8, where the emulsion stability progressively improved with increasing concentration. In general, at lower particle concentrations (e.g., 1% and 2%, *w*/*w*) the CI increased rapidly in a short period time; however, the creaming rate decreased in the following days. For instance, the emulsions stabilized by linseed hull, linseed press-cake, and canola press-cake (at 1%, *w*/*w*), the creaming index reached around 40% after 1 day of storage. This evolution could be attributed to the fact that a part of the particle was not adsorbed into the oil–water interface, generating an instability of the emulsions during storage. 

Consequently, the improvement in the creaming stability observed at high concentrations could indicate that the particle source and concentration could improve the particle-based emulsions against creaming and oiling-off. The dependence of the Pickering stabilizers’ concentration on the creaming behavior was also reported in a previous study [4]. Moreover, an increase in viscosity observed in some samples could prevent the movement of oil droplets. Thus, the improvement of creaming stability could be related to the gel-like network of the Pickering stabilizers. 

## 4. Conclusions

This study demonstrated that agri-food byproducts can be used as raw material to develop successfully edible O/W Pickering emulsions. The results showed that all contain valuable macronutrients and surface-active agents to perform as Pickering stabilizers, such as proteins and fibers. In addition, canola press-cake, camelina press-cake, and linseed hull exhibited a higher antioxidant activity within all the samples evaluated.

The Pickering emulsions stabilized by camelina press-cake, lupin hull, and lupin byproduct samples exhibited extraordinary superior physical emulsion stability in comparison to other agri-food byproducts evaluated in this study, which was confirmed by the high resistance of the emulsions against creaming and no phase separation during 45 days of storage. In addition, the droplet size and stability of the O/W emulsions were strongly influenced by the particle concentration and type of raw material used.

These findings demonstrate that agri-food byproducts are efficient particle-based emulsifiers that can be suitable emulsifiers in industries where food-grade, sustainable, and highly efficient Pickering stabilizers are required.

## Figures and Tables

**Table 1:** Chemical composition of plant-based byproducts (g/100 g).

Parameter	Lupin Hull	Lupin Byproduct	Linseed Hull	Linseed Press-Cake	Canola Press-Cake	Camelina Press-Cake
Protein	17.90 ± 1.08 ^a^	30.80 ± 2.11 ^c^	22.90 ± 1.23 ^b^	40.97 ± 2.97 ^d^	39.80 ± 1.88 ^d^	46.71 ± 2.32 ^e^
Fat	2.07 ± 0.27 ^c^	4.78 ± 0.33 ^d^	0.34 ± 0.08 ^a^	8.71 ± 0.90 ^e^	0.88 ± 0.04 ^b^	0.32 ± 0.02 ^a^
Ash	2.39 ± 0.23 ^a^	5.07 ± 0.47 ^bc^	6.53 ± 0.53 ^d^	5.64 ± 0.61 ^cd^	5.51 ± 0.39 ^c^	4.67 ± 0.42 ^b^
Dietary fiber	67.10 ± 2.19 ^b^	60.59 ± 2.37 ^b^	61.10 ± 2.09 ^b^	40.02 ± 1.21 ^a^	41.60 ± 1.99 ^a^	38.58 ± 1.55 ^a^
Carbohydrates available	10.53 ± 0.82 ^c^	0.11 ± 0.01 ^a^	10.10 ± 0.88 ^c^	41.54 ± 1.91 ^d^	11.20 ± 0.78 ^c^	5.42 ± 0.76 ^b^

Data expressed by mean ± standard deviation, and different letters in the same row indicate a significant difference (*p* < 0.05) between samples.

**Table 2:** Characterization of byproducts from agri-food.

Parameter	Lupin Hull	Lupin Byproduct	Linseed Hull	Linseed Press-Cake	Canola Press-Cake	Camelina Press-Cake
Soluble fraction (%)	19.19 ± 0.91 ^b^	27.92 ± 1.11 ^c^	27.75 ± 2.83 ^c^	16.63 ± 0.95 ^a^	33.56 ± 0.25 ^d^	28.80 ± 0.43 ^c^
Insoluble fraction (%)	80.80 ± 0.91 ^c^	72.08 ± 1.11 ^b^	72.25 ± 2.83 ^b^	83.37 ± 0.95 ^d^	66.44 ± 0.25 ^a^	71.20 ± 0.43 ^b^
Mean size (µm)	1.52 ± 0.28 ^a^	1.06 ± 0.33 ^a^	2.48 ± 0.13 ^b^	3.70 ± 0.93 ^c^	1.26 ± 0.07 ^a^	4.51 ± 0.58 ^c^
Polydispersity index	0.68 ± 0.01 ^d^	0.99 ± 0.39 ^c^	0.72 ± 0.22 ^c^	0.43 ± 0.11 ^b^	0.84 ± 0.38 ^c^	0.08 ± 0.08 ^a^
Form	Irregular	Irregular	Irregular	Irregular	Irregular	Irregular
Zeta potential (mV)	−28.60 ± 2.40 ^b^	−28.15 ± 2.47 ^b^	−25.13 ± 1.56 ^a^	−26.60 ± 4.81 ^a^	−29.10 ± 3.96 ^bc^	−37.53 ± 3.55 ^c^

Data expressed by mean ± standard deviation, and different letters in the same row indicate a significant difference (*p* < 0.05) between samples.

## Data Availability

Data is contained within the article.
